# Experiencing geriatric rehabilitation through the older adult’s eyes: a mixed methods evaluation study of a virtual reality intervention among healthcare professionals

**DOI:** 10.1093/ageing/afag159

**Published:** 2026-06-02

**Authors:** Anne Lubbe, Margriet C Pol, Wim G Groen, Joël van den Berg, Cees Hertogh, Bianca Buurman, Marjon van Rijn

**Affiliations:** Department of Medicine for Older People, Amsterdam UMC Location VUmc, Amsterdam, The Netherlands; Aging & Later Life, Amsterdam Public Health Research Institute, Amsterdam, The Netherlands; Vivium Zorggroep, Naarderheem, Huizen, The Netherlands; Department of Medicine for Older People, Amsterdam UMC Location VUmc, Amsterdam, The Netherlands; Aging & Later Life, Amsterdam Public Health Research Institute, Amsterdam, The Netherlands; Research Group, Occupational Therapy: Technology and Participation, Faculty of Health, Centre of Expertise Urban Vitality, Amsterdam University of Applied Sciences, Amsterdam, The Netherlands; Department of Medicine for Older People, Amsterdam UMC Location VUmc, Amsterdam, The Netherlands; Aging & Later Life, Amsterdam Public Health Research Institute, Amsterdam, The Netherlands; Amsterdam Movement Sciences, Ageing & Vitality, Rehabilitation & Development, Amsterdam, The Netherlands; Department of Medicine for Older People, Amsterdam UMC Location VUmc, Amsterdam, The Netherlands; Aging & Later Life, Amsterdam Public Health Research Institute, Amsterdam, The Netherlands; Research Group Integrated complex care: Faculty of Health, Center of Expertise Urban Vitality, Amsterdam University of Applied Sciences, Amsterdam, The Netherlands; Aging & Later Life, Amsterdam Public Health Research Institute, Amsterdam, The Netherlands; Amsterdam UMC Locatie VUmc, Medicine for Older People, Amsterdam, Noord-Holland, The Netherlands; Aging & Later Life, Amsterdam Public Health Research Institute, Amsterdam, The Netherlands; Department Internal Medicine, Section Geriatric Medicine, Amsterdam UMC Location AMC, Amsterdam, The Netherlands; Department of Medicine for Older People, Amsterdam UMC Location VUmc, Amsterdam, The Netherlands; Aging & Later Life, Amsterdam Public Health Research Institute, Amsterdam, The Netherlands; Research Group Integrated complex care: Faculty of Health, Center of Expertise Urban Vitality, Amsterdam University of Applied Sciences, Amsterdam, The Netherlands

**Keywords:** geriatric rehabilitation, older adults perspective, virtual reality, simulation, older people, qualitative research

## Abstract

**Background:**

Geriatric rehabilitation (GR) aims to optimise functional capacity and social participation in older adults through multidisciplinary care. Healthcare professionals (HCPs) play a key role, and a deeper awareness of patients’ lived experiences may enhance the quality of care. Virtual Reality (VR) offers an innovative way for HCPs to step into the perspective of older adults and experience rehabilitation through their eyes. Recent studies increasingly highlight VR’s potential to foster empathy and communication skills.

**Aim:**

To explore whether a VR movie can enhance HCPs’ understanding and empathy regarding older adults’ experiences during GR.

**Methods:**

In a mixed-methods design we combined structured questionnaires and focus groups. HCPs from four GR organisations viewed a 15-minute VR movie showing the rehabilitation process from the viewpoint of an older adult which was scripted based on earlier studies on this topic. After viewing, participants completed a questionnaire or took part in focus groups discussing their experiences.

**Results:**

In total, 160 HCPs completed the questionnaire and 18 participated in a total of three focus groups. Most participants (85%) reported increased awareness of older adults’ experiences, and 95% indicated that they would recommend the VR movie to colleagues. Thematic analysis identified three key themes reflecting how HCPs, when adopting the older adult’s perspective, perceived the rehabilitation experience: (1) feeling overwhelmed, (2) being dependent and vulnerable and (3) lack of clarity in staff routines. Participants additionally suggested practical improvements in daily care.

**Conclusion:**

The VR movie appears to be an effective educational tool in raising understanding and empathy among HCPs in GR.

## Introduction

Geriatric rehabilitation (GR) aims to restore or maintain the functional independence of older adults following hospitalisation or acute illness. This process requires close collaboration within a multidisciplinary team of physicians, nurses, allied health professionals and support staff [1–3]. The quality of care provided depends not only on clinical expertise, but also on the healthcare professional’s (HCP) ability to understand and empathise with the experiences, preferences and needs of older adults [4]. Previous studies have shown that older adults feel more supported and understood when HCPs actively listen to them, take their concerns seriously and provide appropriate support for the physical, psychological and social challenges they face during rehabilitation [5, 6]. Nevertheless, truly understanding what rehabilitation feels like from an older adult’s perspective remains difficult—especially for those who have never experienced it themselves [7, 8]. Virtual reality (VR) could be a valuable tool to provide experiences that closely resemble real life.

VR has the potential to bridge the gap between healthcare professionals’ perceptions of older adults’ experiences and the realities of those experiences. While VR has traditionally been used to enhance technical training—particularly in surgical or psychomotor domains—there is growing interest in its use for developing non-technical skills, such as empathy, communication and interprofessional collaboration [9, 10]. VR is a valuable tool in healthcare education, providing immersive simulations that allow learners to experience rehabilitation from the perspective of older adults and foster deeper engagement with older adult experiences [11]. Rooted in experiential learning theory, VR supports learning through active participation and reflection [12]. Its effectiveness is further enhanced when the content is closely aligned with the learner’s professional context and prior experience, increasing relevance, retention and the transfer of learning to clinical practise [13]. VR allows users to step into another person’s shoes in ways traditional educational tools cannot [11]. However, it remains unclear to what extent such immersive educational interventions can enhance HCPs’ understanding of older adults’ experiences during geriatric rehabilitation.

The aim of this study is to explore whether using a VR movie as an educational tool can enhance HCPs’ understanding of older adults’ experiences during geriatric rehabilitation. Specifically, we examine whether the VR movie can increase awareness and empathy among HCPs.

## Methods

### Study design and setting

We used a mixed methods design. To gather experiences from a large sample of HCPs, the research team collected data through questionnaires (including both closed and open-ended questions). To gain a more in-depth understanding, we conducted focus groups. Representatives from different disciplines participated in both the questionnaire and the focus groups. Participating organisations are affiliated with the University Network of Care Organisations for Older Adults (UNO) Amsterdam. A convergent design was used, with quantitative and qualitative data collected independently and integrated during analysis to yield a comprehensive understanding of the findings.

### Participants

Between April and June 2024, HCPs working in four GR organisations [A, B (location 1 & 2), C (location 1 & 2) and D] were invited to participate. The inclusion criteria were: proficiency in Dutch, including adequate verbal, reading and writing skills.

Within each organisation, a designated contact person informed HCPs about the study via email, posters and flyers. HCPs from the organisations could voluntarily register via email to watch the VR movie and complete the questionnaire. Purposive sampling was applied only for the focus groups. Focus groups were conducted at organisations A, B and C by A.L.L and J.J.C.B. The purpose of the sampling strategy was to ensure representation of different HCP disciplines involved in geriatric rehabilitation. At each location where a focus group took place, we aimed to include a diverse mix of disciplines to reflect GR's multidisciplinary nature. Years of professional experience were not used as a selection criterion. In organisation D, a focus group was not feasible because of the high workload.

### Data collection

Participants watched a 15-minute VR movie titled ‘Geriatric rehabilitation from the perspective of the rehabilitation patient’.

### VR movie titled ‘Geriatric rehabilitation from the perspective of the rehabilitation patient’

**Figure 1:**
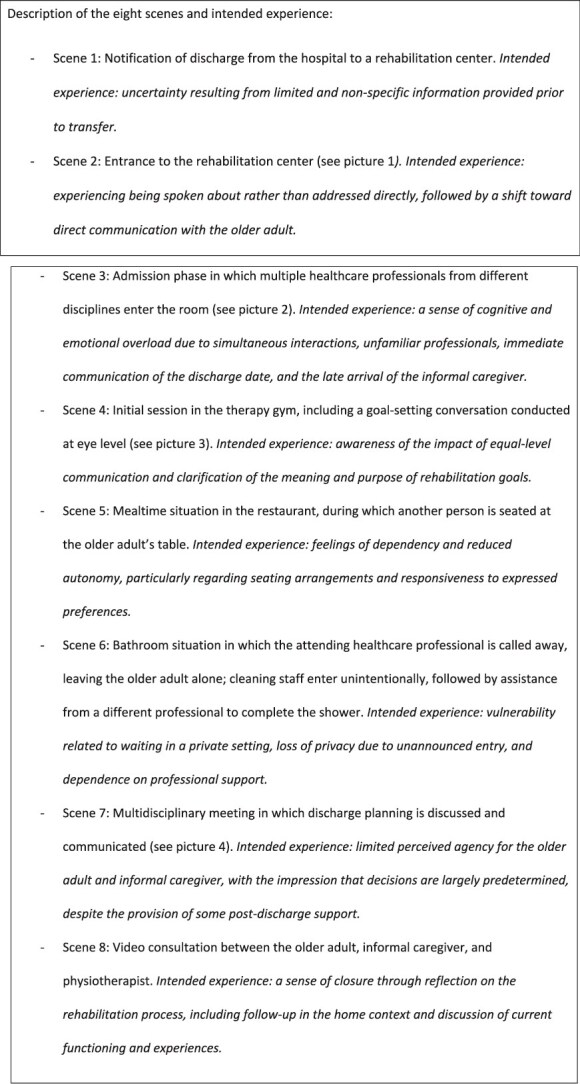
The content of the VR movie ‘Geriatric rehabilitation from the perspective of the rehabilitation patient’ + description and intended experience of the eight scenes of the VR-movie.

The script drew on findings from a scoping review and an interview study exploring older adults’ experiences in GR settings [5, 6]. The movie follows an older adult throughout the entire rehabilitation journey—from hospitalisation, to the geriatric rehabilitation centre, to returning home. Guided by the principles of experiential learning, the research team designed the VR movie to translate research findings into an accessible, practise-oriented learning experience [12]. The script was co-created by researchers and reviewed by older adults, HCPs and managers. VR Gorilla filmed the movie in 3D, 360° format (VR Gorilla®), with volunteer actors who work in geriatric rehabilitation centers. A description of the VR-movie and a selection of screenshots from the VR movie is presented in Figure 1 to illustrate what participants experienced.

**Picture 1:**
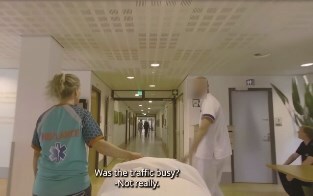
Entering the rehabilitation centre; HCPs talk to each other but not to the older adult.

**Picture 2:**
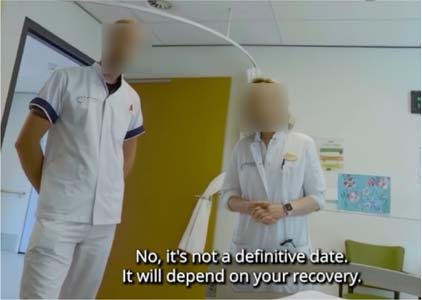
HCPs stand next to the patient's bed, discussing the discharge date on the first day of admission.

**Picture 3:**
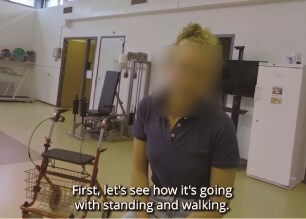
Experiencing the difference when a HCP sits next to the older adult.

**Picture 4:**
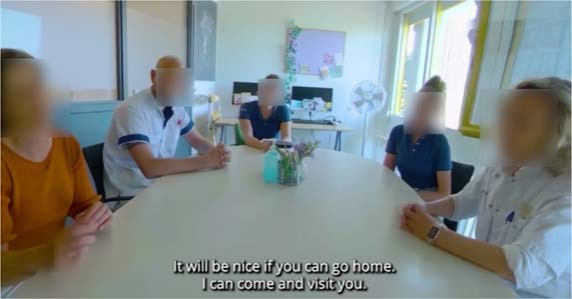
Experiencing the entire HCP team around the older adult, together with the informal caregiver, discussing discharge plans.

After watching the VR movie, participants completed a questionnaire with five closed questions, consisting of 5-point Likert scale items (strongly agree, agree, neutral, disagree, strongly disagree), allowing them to explain their chosen answer, and three open-ended questions (Appendix 1). Building on a questionnaire developed for a previous study by colleagues from our institute [14], we adapted it to the specific context of GR. The questionnaire investigated the HCPs' awareness of the rehabilitation process from the older adult's perspective, their perception of the care received and their willingness to recommend the VR movie to colleagues. We pilot-tested the questionnaire with students, researchers and HCPs from our department. Additionally, the focus groups lasted two hours and we conducted them in different organisations. A topic list (Appendix 2) based on the questionnaire guided the focus group, moderated by A.L.L., J.J.C.B. took notes, (both female).

A.L.L. is physiotherapist at one of the centers where a focus group was conducted; however, no direct colleagues of A.L.L. participated in that focus group. In the other two focus groups, there were no prior professional or personal connections between the moderator and the participants.

### Data analysis

Quantitative data from closed questions were analysed using IBM SPSS Statistics version 28. We calculated the frequency distributions of responses for each item to provide an overview of participants' responses. Using these data, we analysed the distribution of opinions and attitudes across the different statements. Descriptive explanations of the closed questions were provided to illustrate the questionnaire results. We analysed the qualitative data from open-ended questions and focus groups to identify overarching themes in participants' responses. The focus groups were recorded and transcribed verbatim. Data were analysed using thematic analysis [15]. For the focus groups, in (phase 1 familiarisation with the data, the transcripts were read repeatedly by A.A.L and J.J.C.B.). In phase 2 (generating initial codes), Inductive open coding was applied. Coding of the focus group transcripts was performed independently by A.L.L. and J.J.C.B., in accordance with the principles of thematic analysis. In (phase 3: searching for themes), codes were systematically clustered into potential themes which were then reviewed (phase 4: reviewing themes), redefined in collaboration with the research team (phase 5: defining and naming themes). The final themes were written up (phase 6: producing the report). Transcripts of the focus groups were not returned to participants for member-check. The **open-ended questionnaire responses** were analysed separately. These responses were reviewed by the research team and discussed until consensus was reached on the interpretation and categorisation of the data. The results of the questionnaires were not shared with participants.

We used MAXQDA2022 for data management and analysing the data. Triangulation could be found in the three types of data source methods (closed questions, open questions and focus groups) to gain a comprehensive understanding of phenomena [16].

### Ethics

This research was approved by the Medical Ethics Review Committee of Amsterdam UMC (2023.1037). Detailed information about the study was provided to participants through an information letter, outlining the purpose and procedures. To ensure the quality and transparency of this research, the Consolidated Criteria for Reporting Qualitative Research (COREQ) checklist was used (Appendix 3).

## Results

A total of 160 HCPs watched the VR movie and participated in the study by filling out a questionnaire after watching the VR movie. Eighteen HCPs participated in a total of three focus groups. All data were collected in the rehabilitation centers where the HCPs are working.

### Questionnaire

A total of 160 participants completed the questionnaire, comprising 90% female and 10% male participants (Table 1). The ages ranged from 17 to 64 years, with a mean age of 38 years. The participants represented a variety of professions, including nurses, physicians, paramedics, healthcare assistants, facility staff and others. Their work experience ranged from 0 to 35 years, with a mean of 9 years. Based on the number of completed questionnaires, approximately 83% of the staff present per day over the six testing days participated in this study.

**Table 1:** Demographic characteristics participants’ questionnaire.

	*N* = 160	%
Sex		
Male	16	10.0
Female	142	90.0
Mean age [range (SD)]	38.0	17–64 (14.6)
Profession		
Nurse	53	33.1
Physician	11	6.9
Paramedics	54	33.8
Health care assistant	11	6.9
Facility staff	11	6.9
Manager	6	3.8
Other	14	8.8
Work experience GR in mean [range (SD)]	9	0–35 (9.6)
Missing^a^	45	28.1

Other are for example informal care, medical secretary and nutritional assistant.

^a^The 45 missing values for years of work experience are mainly attributable to participants who were still in training and those without direct contact with rehabilitation patients.

### Closed questions

**Figure 2:**
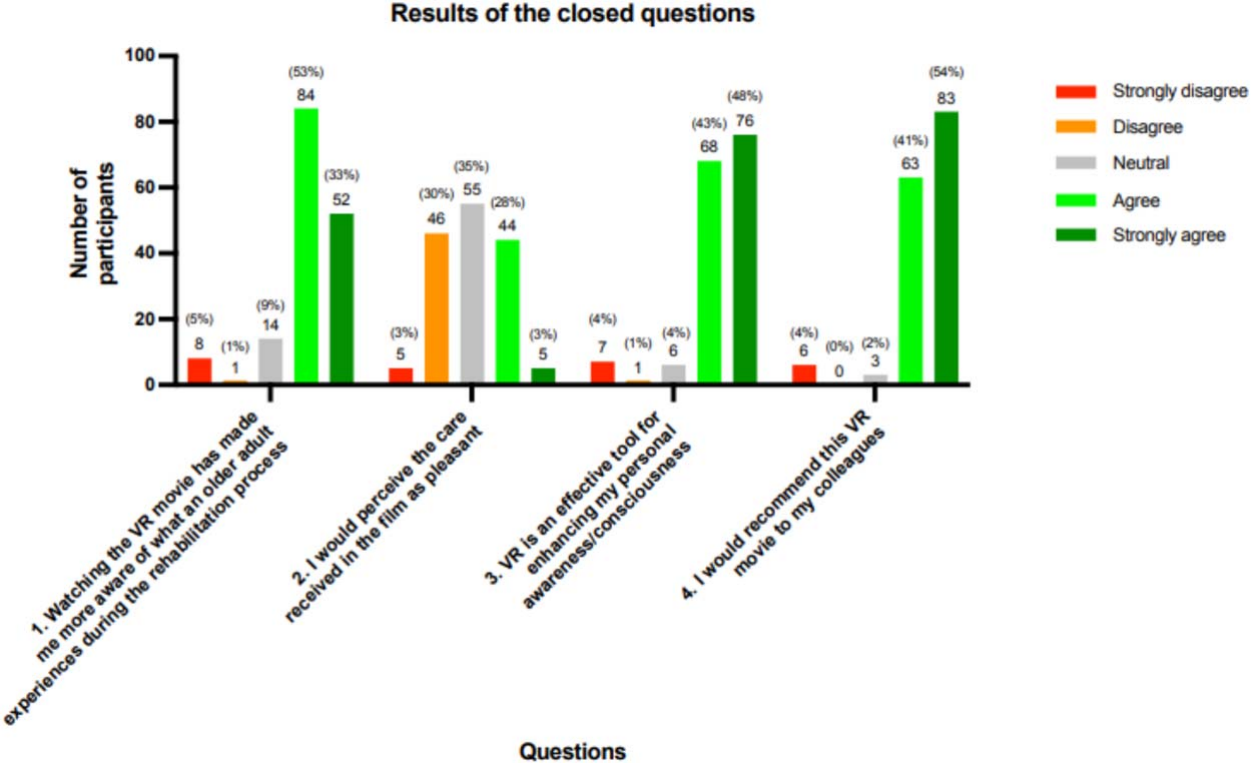
Results of the closed questions.

The results of the closed questions in terms of percentages and frequencies are presented in Figure 2.

#### Awareness of the rehabilitation process from the older adults’ perspective

A majority of the participants indicated that watching the movie increased their awareness of older adults’ experiences during the rehabilitation process, with 53% agreeing and 33% strongly agreeing with the statement (total agreement of 86%). Participants reported gaining new insights into the rehabilitation process through the VR movie, which led to a greater appreciation for the challenges and emotions of older adults. For them, it contributed to a better understanding of the older adult's perspective.

### Perception of perceived care

About one-third (35%) of the participants were neutral about the perceived care in the movie. Among the remaining 60%, approximately half found the perceived care pleasant, with 28% agreeing and 3% strongly agreeing, while the other half found it unpleasant, with 30% disagreeing and 3% strongly disagreeing. The VR movie evoked strong emotional reactions among the HCPs. Many found the film confronting, especially as it highlighted patients' vulnerability and the lack of personal attention and involvement. One participant described it in the option to provide an explanation as:

*It's strange that you seem to end up in a quite rigid system where you seem to have little input yourself. (P. 69)*

#### Enhancing personal awareness

A majority of the participants found VR an effective tool for enhancing their personal awareness, with 43% agreeing and 48% strongly agreeing with the statement. Only 5% of the participants disagreed. Many participants praised the VR experience for its realistic portrayal of the rehabilitation process. This insight helped participants better empathise with the situation of older adults, leaving a deep impression on them.

#### Willingness to recommend the VR movie to colleagues

The willingness to recommend the VR movie to colleagues was very high, with 41% of participants agreeing and 54% strongly agreeing with the statement. The participants were positive and enthusiastic about the learning experience, often suggesting that everyone should have the opportunity to experience it.

### Overall appreciation

The average rating for the VR movie on a ten-point scale was 8.3 (median 8.0, IQR 1.0), with a range of 6.0 to 10.0.

Subgroup analyses were performed to assess whether responses to the closed questions differed according to age, years of professional experience, or profession. No statistically significant differences were observed across these subgroups, indicating that these factors did not influence the responses (Appendix 4).

### Open-ended questions

The open-ended questions concerning participants’ initial reactions, the most impactful moment in the VR film, their evaluation of the quality of guided reflection (GR), and the actions they intended to approach differently the following day, yielded responses that closely aligned with those expressed in the focus groups. Consequently, these responses were incorporated into the focus groups' findings.

### Focus groups

Three focus groups consisting of a total of eighteen HCPs from different disciplines (Table 2) were conducted. During each focus group, only staff members from the respective location were present, together with A.L.L. and J.J.C.B.; no other individuals attended. The focus groups lasted between 1.5 and 2 hours. The participants represented the multidisciplinary team involved in the rehabilitation of an older adult.

**Table 2:** Demographic characteristics of the participants of the focus groups.

Focus group	Participant	Age	Sex	Profession	Work experience GR in years
1	1	18	F	Nurse	0.2
	2	51	F	Elderly care physician	12
	3	31	M	Spiritual counsellor	0.2
	4	31	F	Welfare and informal care coordinator	6
	5	46	F	Nurse	10
	6	55	F	Physiotherapist	12
2	7	32	F	Occupational therapist	9
	8	42	F	Nurse	22
	9	53	F	Nurse	33
	10	49	F	Nurse	32
	11	30	F	Physiotherapist	8
	12	52	F	Nurse	33
	13	23	F	Nurse	3
	14	37	F	Nurse	7
3	15	55	F	Spiritual care practitioner	4
	16	40	F	Physician assistant	15
	17	40	F	Team coach nurse	10
	18	24	F	Clinical psychologist	2

The focus group findings are presented from a dual perspective. On the one hand, they reflect the perspective of older adults as depicted in the VR movie and interpreted by the HCPs; on the other hand, they capture the HCPs’ own reflections on their professional practise following the immersive experience. The analysis resulted in three themes: [1] feeling overwhelmed, [2] being dependent and vulnerable and [3] lack of clarity in staff routines. The coding tree is provided in Appendix 5.

#### Feeling overwhelmed

HCPs described the experience of watching the VR movie as overwhelming on multiple levels, including the intensity of the rehabilitation process and the presence of so many new people around them.

*It all comes to you. I felt like I had no say in what was happening. One person comes in, then another. It’s quite a lot. Everyone talks about you while you’re just sitting there. (P4)*

This awareness prompted HCPs to reflect critically on their own behaviour. Several HCPs began to question whether their actions, though done with good intentions, actually felt respectful or comforting from the older adults' perspective.

*Speaking for myself, I think that when I’m standing at someone’s bedside, I’m doing well — I’m calm, I explain everything. But that’s what I tell myself. Now, seeing it from another perspective, I wonder if it’s really as good as I thought. (P15)*

In addition to questioning whether this reflects appropriate patient care, HCPs also recognised the familiar routines and daily working practises embedded in the scenario.

In addition to the confronting aspects, the movie also helped professionals realise the power of small gestures. HCPs recognised the impact that their presence can have on an older adult.

*And suddenly you think: for me, it’s not such a big deal, but to them it can feel like I have spent hours listening to their story. Just sitting down and really listening for five minutes can already bring relief. (P14)*

Finally, HCPs also reflected on the overwhelming nature of the older adult experience and the realism conveyed by the VR movie, describing it as a mirror held up to their own practise.

*Yes, now you’re fully immersed. I do think it adds something. I think otherwise, you’d feel less uncomfortable. And it would feel more distant – maybe then the nurse at the table wouldn’t have felt as unpleasant. (P16)*

### Being dependent and vulnerable

Most HCPs stated that the experience gave them a deeper understanding of what it feels like to be an older adult undergoing rehabilitation. Many described an intense awareness of dependency. Lying in bed, unable to move freely and relying entirely on others made them feel dependent—a feeling they had underestimated in their daily practise.

*Yes, they are dependent, so in a way you just hope they’ll be treated well. But they are completely at our mercy, and I find that quite confronting when you see it like that. (P14)*

In the older adult role, HCPs felt vulnerable and exposed as HCPs entered their personal space, leaving little to no room for older adult input.

HCPs reflected on their role, considering their attitudes toward older adults and how these attitudes influence the direction in which they guide individuals.

*I think what’s missing is both time and the awareness that this is how it should be done. There’s this perception that it takes more time, even if that might not actually be the case — but the idea that it does makes a difference. (P16)*

HCPs also hear themselves saying, ‘I understand you.’ However, when they find themselves in the role of the older adult, they experience a sense of questioning, ‘What do you understand?’ This is something they practise, and one of the things they would like to remove from their automatic responses.

*Interestingly, during our training, we were taught not to say ‘I understand how you feel’ anymore. Because you don’t really know what someone is going through at that moment. You can try to imagine it, but I wouldn’t say it with every situation anymore. (P1)*

#### Lack of clarity in staff routines

HCPs said as an older adult in the VR, you are unaware of the routines within GR and why everyone acts as if this is normal, leading to a sense of confusion about what is actually happening to you.

*Why doesn't someone explain what they are doing? So I take that with me, the full transparency of the staff in their actions. (P. 3)*

Several HCPs shared that the movie made them realise how easily they slip into routines that feel completely normal to them. However, it is often a first-time experience for the older adult. The movie helped them see that GR, while part of their work as staff, is a major life event for older adults, often following a sudden serious illness. Through this new perspective, professionals became more aware of the need to explain what is happening and acknowledge how unfamiliar the situation can be for the older adult. This highlights the importance of clear explanation and communication, which may be overlooked in routine practise and can otherwise lead to miscommunication about what older adults can expect.

*Things feel so automatically ‘normal’ to us — but I’d like to take with me the idea that it’s not normal at all. We do this every day, but for the people who come here — it might be just once in their life. Something has happened to them, and it’s not normal. So, like you said, we need to explain much more, to name what’s happening, that kind of thing. (P2)*

Many professionals described how the VR movie made them more aware of routines they usually perform and helped them recognise that these actions might not feel right from the older adults' point of view.

*I think it’s also about awareness. […] I believe you're already taking a big step forward when you become really conscious of it — instead of just acting on autopilot. Because that’s how it often goes during a day like that: so many things you do automatically. (P2)*

HCPs indicate that much happens on autopilot, that perhaps the response is not truly listened to, but rather absorbed into daily routine.

*When I take someone to the restaurant, I often ask, ‘Is that okay?’ — but I ask it without really listening. Even if they said no, I probably wouldn’t notice. (P14)*

HCPs indicated that the VR movie made them more aware of the importance of truly seeing the individual. They expressed a desire to approach each situation with an open mind, taking into account the older adult's perspective rather than focusing solely on time and efficiency. This underscores that communication and emotions are fundamental to establishing a constructive therapeutic relationship.

*But also being present for someone’s emotions — when someone is just feeling sad, for instance. (P6) That you sit down with them. (P5) Yes, to sit down with them and say: it’s okay to feel this way, a lot has happened. Just showing a bit of understanding. And if you can’t laugh right now, that’s okay too. (P6)*

### Practical improvements in daily care

During the discussions, participants also formulated *implications for practise*.

Several HCPs mentioned that they want to change their approach to older adults by addressing them more at eye level. Others indicated that they want to use more humour to put older adults at ease. They recognise the need to rely not only on standard procedures but also to be flexible and responsive to each older adult's needs. From the older adult’s perspective, such small, practical adjustments may result in meaningful improvements in care experience.

On the other hand, HCPs emphasised the ongoing challenges associated with personal care, such as time constraints and a lack of resources. While it made them more aware of older adults' needs, many indicated that implementing changes remains challenging due to structural limitations within the healthcare facility. Participants repeatedly emphasised the need for sufficient time and space to deliver quality care, describing this as a constant struggle. HCPs suggested that sharing these insights with management could be a first step toward addressing these challenges at an organisational level.

*Yes, you want to get your work done, but in the meantime, you also want to do it as well as possible. (P5)*

Therefore, change will also need to come from management. Involving health insurers and policymakers in the VR movie was also suggested to better understand the challenges in care and stimulate policy improvements.

*That management sees this too. (P5) So maybe we shouldn’t just wait for that to happen, but actively try to spark it. Because I really do think it’s important. When I look at it like this, I think: this person and that person should really see this too. And I don’t mean that in a negative way — I just think it would really be helpful. (P4)*

In addition to its potential value for management, the VR movie may also serve as an educational tool. The VR movie was considered valuable for both practising HCPs and students. However, HCPs indicated a preference for viewers to have some prior healthcare experience before engaging with the VR movie. Such experience was considered important to ensure that viewers have a realistic understanding of everyday practise, rather than an idealised educational perspective, allowing the impact of the VR experience to be more fully realised.

Participants reported that the VR experience felt highly realistic and preferred it to a flat-screen display. They suggested that this type of training should become a mandatory component of professional development, with dedicated time allocated by management. Participants emphasised the importance of continuous awareness and reflection, noting that repeated exposure to VR experiences could support sustained improvements in care practises. In addition to viewing the VR movie, participants highlighted the value of team-based reflection and discussion. Finally, participants suggested that VR simulations could be adapted to different care contexts, such as psychogeriatric wards, to enhance relevance and impact.

*I do think you get more out of it when you understand how things work. If you know the routines, you can gain more from it. (P17) I think it’s more confronting when you know how you work yourself — you notice more than when you come in completely fresh. (P15)*

## Discussion

In this study, we explored whether a VR movie could serve as an educational tool to enhance HCPs’ understanding of older adults’ experiences during GR. Specifically, we investigated whether VR could foster awareness and empathy among HCPs.

The quantitative findings showed that watching the VR movie increased HCPs' awareness. The qualitative analysis revealed that HCPs perceived the rehabilitation process as overwhelming when stepping into the older adults’ perspective. HCPs mentioned and reflected on dependency, vulnerability and the dominance of routine-driven practises in daily care. These reflections often led to concrete suggestions for improving their own professional behaviour.

Similar effects have been observed in studies using VR to experience the perspective of someone else, particularly regarding the outcome of empathy. In a study evaluating the VR intervention Through the D’mentia Lens [17], informal caregivers reported a deeper understanding of the lived experiences of people with dementia. The VR movie enabled them to better interpret the perceptions and emotions of the person with dementia, and increased caregivers’ confidence in their care role. [17] Furthermore, Dutton *et al*. [18] demonstrated that VR simulations positively influence HCP behaviour by enhancing knowledge and empathy toward patients with sensory impairments. In their study, HCPs experienced conditions common in older adults—such as hearing loss and macular degeneration. Significant improvements were found in knowledge scores and empathy [18], consistent with our findings that VR can improve empathy among HCPs. Similarly, Liu *et al*. [19] used the Kiersma-Chen Empathy Scale to assess changes in empathy among undergraduate nursing and occupational therapy students following immersive VR-assisted experiential learning. Empathy scores increased significantly, and focus groups confirmed enhanced empathy toward older adults with cognitive impairment. Despite differences in participant groups and clinical contexts, these studies all show that adopting the perspective of another person through VR increases empathy among both HCPs and informal caregivers [20]. This shows the potential of VR as a valuable educational tool, also in geriatric care. While the alternative perspective is conveyed through the different VR scenarios, determining how this can be translated into practical behavioural change and integrated into clinical practise represents an important direction for future research.

The VR film was developed to enhance the patient-centred quality of GR. Although there are multiple guidelines for quality of care and GR [21, 22], perspectives on quality and what is (most) important vary among individuals. This variety makes it difficult to interpret and compare assessments of care quality across different perspectives [23–26]. Clarifying the meaning and scope of the patient perspective is therefore essential for its effective integration into quality assessments. Experiencing the patient’s viewpoint first-hand—such as through VR—may facilitate imaginative engagement, foster empathy and support the development of therapeutic relationships.

Furthermore, communication forms the foundation of a strong relationship between HCPs and older adults. Improved communication within healthcare teams has been associated with better quality of care and higher patient satisfaction [27]. However, often mentioned suggestions for improving quality of care, are related to communication [28], and therefore seem to remain a challenge in health care. Reasons for communication shortcomings are most closely linked to workload and time constraints, as well as the need for communication training [27]. Experience communication from a patient perspective with this VR may contribute to improve awareness for communication for HCPs and reflecting on their own communication. The opportunity to experience various forms of communication in the VR movie makes it a learning method that teaches the importance of effective communication through experience. Beyond strengthening communication, future research could examine whether VR movie may also facilitate the integration of the older adult perspective into care and promote its transparent discussion within the team.

### Implications for education

As implied in the results, the focus groups provided concrete suggestions for practical improvements in daily care, practise-based learning and other implications for education. Participants suggested that VR training should become a mandatory part of the training curriculum in health care organisations, with repeated exposure for improvement in practise, especially when the VR movie is watched together with the multidisciplinary team, with the aim of improving the quality of care. Although it has been suggested, that repeated exposure to the same VR movie may lead to a diminishing impact, a study found that repeated viewing of VR movies did not significantly reduce viewers' attention or load, suggesting that the immersive nature of VR may sustain high engagement even with repetition [29]. In addition, for successfully implementing a teaching package with the VR movie, an experienced trainer who can guide the debriefing is needed. This requires technical VR knowledge, didactic and content skills, as well as time and money. For optimal learning, it is crucial that students are well guided in evaluating and reflecting on the VR movie [30]. Without the proper guidance, students may struggle to achieve learning goals and utilise the full potential of VR as an educational tool.

Future research is needed to determine whether increased awareness and empathy translate into real benefits for older adults in real-world practise. For example, HCPs could be asked to document the improvements they intend to implement after first viewing the VR movie, followed by observation or evaluation of their behaviour in practise to assess whether meaningful change occurs. It is important to emphasise the value of small, practical adjustments in daily routines—such as sitting down with older adults and providing clear information—which may contribute to reduced workload and improved job satisfaction.

Further research should specifically examine whether behavioural change arises from the knowledge gained through exposure to the older adult’s perspective. An important question for future studies is whether the experience of adopting the older adult’s perspective remains salient for HCPs over time, and how this influences practise on the work floor.

### Strengths and limitations

The strength of this study lies in the use of a mixed-methods approach. By combining quantitative data from the closed questions of the questionnaire with qualitative insights derived from the open-ended questions and focus group discussions, the study provides a comprehensive understanding of the impact of a VR movie on the quality of GR from the perspective of the older adult. The open-ended questions and focus group discussions offered deeper insight into the participants' experiences. By using a questionnaire, we could ask a large group of HCPs about their experiences. However, adding the focus group enabled us to elaborate on a comprehensive understanding of the potential effects on awareness and empathy and how they might translate to practical improvements. In addition to the use of both qualitative and quantitative data collection methods, the inclusion of participants from multiple professional disciplines enhances the generalisability of the findings within GR. A further advantage of the VR movie is that all participants undergo the same experience, ensuring a comparable frame of reference and facilitating meaningful discussion and shared learning.

The study also has several limitations that should be acknowledged. First, the impact of the VR movie is only observed immediately after watching it. The study focuses on the immediate effect of participants' awareness and attitudes. Therefore, this study cannot provide insights into the actual impact on patient care.

Second, the focus group part of the study may have been at risk for performance bias. That is, participants' responses may have been influenced by their awareness of being observed or their desire to provide socially desirable answers, which could affect the reliability of the findings. For instance, healthcare professionals might have overstated their emotional engagement or empathy toward the vulnerable older adult depicted in the VR scenario to align with expected social and professional norms. This risk may have been amplified by the face-to-face discussions with colleagues, which could have encouraged participants to express socially and professionally desirable attitudes.

Third, the questionnaire we used was not formally tested on psychometric properties, and are therefore unknown. However, we used the questionnaire our colleagues developed to evaluate their VR [14] as a basis for adapting it slightly to our context. Furthermore, to gain a deeper understanding, we gathered additional insights from HCPs by conducting focus groups, strengthening the outcome.

Fourth and finally, within this VR simulation there was no possibility for active response during viewing, which may limit the extent to which real-world interaction between individuals can be simulated. However, in this context, this apparent ‘limitation’ may have reinforced feelings of dependency, being spoken over rather than spoken with, and loss of autonomy. Although moments of silence were intentionally incorporated into the VR movie to allow for potential responses, observational data indicated that participants did indeed attempt to respond during these pauses. Nevertheless, the current VR technology did not yet allow the simulation to adapt to or engage with participant interaction.

More generally, VR is a relatively new and rapidly evolving medium for education and training. Since the development of the VR movie and the execution of this study, technological advances have already enabled interactive VR experiences. Incorporating such interaction could further enhance realism and deepen the experiential impact of VR-based simulations.

It is possible that the immersive nature of a first-time VR experience contributed to the reported feelings of being overwhelmed. For further research with VR it is relevant question a question about whether participants have had prior exposure to VR technology, to assess whether the medium itself may have been experienced as overwhelming.

## Conclusion

The VR movie appears to be an effective tool for raising awareness and empathy among HCPs regarding older adults' experiences in GR. Further research should investigate its potential for (long term) change in daily practise.

## Supplementary Material

aa-25-3437-File002_afag159
