# Supplementary material for: Experiencing geriatric rehabilitation through the older adult’s eyes: a mixed methods evaluation study of a virtual reality intervention among healthcare professionals
Source: Age Ageing. 2026 Jun 2;55(6):afag159. doi: 10.1093/ageing/afag159 (PMC13229252; doi:10.1093/ageing/afag159)
Supplement: aa-25-3437-File002_afag159 [file aa-25-3437-file002_afag159.docx]

# Appendices

**Appendix 1. Questionnaire VR movie: Geriatric rehabilitation from the older adults perspective**
“The data will be used for research purposes and will be processed and published anonymously and without being traceable to individuals.”

*By completing this evaluation form, I give permission for my data to be used for research purposes and publication.*

- Yes, I give my permission
- No, I do not give my permission

### Personal Information:

- Age:
- Role/Profession:
- Years of experience in geriatric rehabilitation (if applicable):
- Gender: m / f / x

### Open-ended questions

1. Please describe your initial reaction to the movie in a few sentences.

2. Which moment in the movie affected you the most, and why?

3. Based on this VR experience, what do you consider to be quality in geriatric rehabilitation from the patient's perspective? You may provide multiple examples.

4. What is one thing you will do differently tomorrow after watching the movie?

### Closed questions

1. Watching the movie made me more aware of what a patient experiences during rehabilitation.
   - Strongly disagree
   - Disagree
   - Neutral
   - Agree
   - Strongly agree

Additional comments:

1. I would have experienced the care shown in the movie as pleasant.
   - Strongly disagree
   - Disagree
   - Neutral
   - Agree
   - Strongly agree

| The aspects of care I found pleasant: | The aspects of care I did not find pleasant: |
| --- | --- |

1. I believe virtual reality is a good tool to enhance my personal awareness.
   - Strongly disagree
   - Disagree
   - Neutral
   - Agree
   - Strongly agree

Additional comments:

1. I would recommend this VR movie to my colleagues.
   - Strongly disagree
   - Disagree
   - Neutral
   - Agree
   - Strongly agree

Additional comments:

1. My rating for the VR movie “Geriatric Rehabilitation from the Patient’s Perspective” [0–10]: …

**Thank you for completing this questionnaire. If you have any other comments, suggestions, or questions, please share them below:**

## Appendix 2. Topic list focus group

**Introduction**

- Welcome participants and introduce the purpose of the focus group.

**Background Information**

- Collect demographic data (age, gender, role, and years of experience in geriatric rehabilitation).
- Let’s start with a short round of introductions. Please tell us who you are and what your role is in geriatric rehabilitation.
- Icebreaker: What is your personal strength in working within geriatric rehabilitation?

**Experience with the VR movie**

- How did you experience the VR movie?
- How effective did you find the VR movie in helping you empathise with the perspective of the rehabilitation patient and their rehabilitation process?
- Which part of the movie stood out to you the most?
- In your opinion, what matters most to the rehabilitation patient? What defines quality of care from their perspective?

**Changes in Daily Practice**

- Can you share examples of how the VR movie might influence your approach to daily care?
- When you’re at the patient’s bedside tomorrow, do you think you’ll approach things differently?
- How can these changes be sustained in the long term?

**Potential Applications**

- Do you think this VR movie could be used in the education of (future) healthcare professionals?
- What could a future training program based on this VR movie look like?
- What kind of information would you need beforehand to make the most of watching the movie?

**Closing**

- Thank the participants for their time and valuable input.
- Are there any final thoughts or questions?

**Schedule**

**13:30–13:45** → Welcome with coffee/tea, introductions & icebreaker

- Round of introductions + making name tags
- Share your expectation in one word
- Name your personal strength in GR
- Collect on paper: age, years of experience in GR, role, gender

**13:45–14:00** → Watching the VR movie

**14:00–14:15** → First reactions

- Ask participants to write their initial reactions on a large sheet of paper
- Use these as talking points in the following discussion

**14:15–15:15** → Open group discussion

- Let participants lead the discussion as much as possible
- Guide with summaries and open-ended questions
- Pay attention to and address non-verbal cues

**15:15–15:30** → Wrap-up

## Appendix 3 COREQ (COnsolidated criteria for REporting Qualitative research) Checklist

| **No** | **Topic** | **Guide question/description** | **Answer** | **Page no.** |
| --- | --- | --- | --- | --- |
| Domain 1: research team and reflexivity | | | | |
| *Personal characteristics* | | | | |
| 1. | Interviewer/facilitator | Which author/s conducted the interview or focus group? | AL and J.J.C.B. conducted all focus groups | Data collection  Page 6 |
| 2. | Credentials | What were the researcher’s credentials? E.g. PhD, MD | MSc | Title page |
| 3. | Occupation | What was their occupation at the time of the study? | A.L.L. PhD student and physiotherapist, J.J.C.B. master student | Title page |
| 4. | Gender | Was the researcher male or female? | Both, female | Data collection  Page 6 |
| 5. | Experience and training | What experience or training did the researcher have? | The researchers followed several qualitative research courses and courses on interviewing techniques. | N/A |
| *Relationship with participants* | | | | |
| 6. | Relationship established | Was a relationship established prior to study commencement? | Yes, in one of the focus groups colleagues of A.L.L. participated, but not the direct colleagues. | Data collection  Page 7 |
| 7. | Participant knowledge of the interviewer | What did the participants know about the researcher? e.g. *personal goals, reasons for doing the research* | The participants knew the researchers’ affiliation and profession. | N/A |
| 8. | Interviewer characteristics | What characteristics were reported about the interviewer/facilitator? e.g. *Bias, assumptions, reasons and interests in the research topic* | No further characteristics were reported than researcher’s affiliation and profession. | N/A |
| Domain 2: study design | | | | |
| *Theoretical framework* | | | | |
| 9. | Methodological orientation and Theory | What methodological orientation was stated to underpin the study? e.g. *grounded theory, discourse analysis, ethnography, phenomenology, content analysis* | Thematic Analysis | Data analysis  Page 7 |
| *Participant selection* | | | | |
| 10. | Sampling | How were participants selected? e.g. *purposive, convenience, consecutive, snowball* | Purposive sampling for the focus groups, for the diversity of disciplines | Participants  Page 3 |
| 11. | Method of approach | How were participants approached? e.g. *face-to-face, telephone, mail, email* | The organisations’ contact person informed HCPs via email, posters, and flyers. | Participants  Page 3 |
| 12. | Sample size | How many participants were in the study | 18 participants for the focus groups, 160 participants for the questionnaire | Results  Page 8 |
| 13. | Non-participation | How many people refused to participate or dropped out? Reasons | There were no drop outs. Refused HCP of the questionnaire, should be somewhere around 15-20% | Results questionnaire  Page 8 |
| *Setting* | | | | |
| 14. | Setting of data collection | Where was the data collected? e.g. *home, clinic, workplace* | In the rehabilitation centres | Results  Page 8 |
| 15. | Presence of non-participants | Was anyone else present besides the participants and researchers? | There was no one else present. | Results focusgroep  Page 12 |
| 16. | Description of sample | What are the important characteristics of the sample? e.g. *demographic data, date* | Participants were HCPs in the GR centres | Results table 2  Page 12 |
| *Data collection* | | | | |
| 17. | Interview guide | Were questions, prompts, guides provided by the authors? Was it pilot tested? | A topic guide was developed by two authors (A.L.L and J.J.C.B.). | Data collection  Page 7 |
| 18. | Repeat interviews | Were repeat interviews carried out? If yes, how many? | There were 3 focus groups, with the same topic list. | Results focus groups  Page 12 |
| 19. | Audio/visual recording | Did the research use audio or visual recording to collect the data? | Audio recordings were used. | Data analysis  Page 7 |
| 20. | Field notes | Were field notes made during and/or after the interview or focus group? | Yes, some notes were made during the focus groups | Data collection  Page 7 |
| 21. | Duration | What was the duration of the interviews or focus group? | 60-120 minutes | Results focus group  Page 12 |
| 22. | Data saturation | Was data saturation discussed? | We have tried to reach data saturation. | N/A |
| 23. | Transcripts returned | Were transcripts returned to participants for comment and/or correction? | Transcripts were not returned. | Data analysis  Page 8 |
| Domain 3: analysis and findings | | | | |
| *Data analysis* | | | | |
| 24. | Number of data coders | How many data coders coded the data? | Both AL and J.J.C.B have separately coded all transcripts. Thereafter all codes were thoroughly discussed with the research team during data analysis | Data analysis  Page 7 |
| 25. | Description of the coding tree | Did authors provide a description of the coding tree? | Yes, there is a coding tree | Results focus group  Page 12 |
| 26. | Derivation of themes | Were themes identified in advance or derived from the data? | Derived from the data | Data analysis  Page 7 |
| 27. | Software | What software, if applicable, was used to manage the data? | Transcripts were coded in MAXQDA | Data analysis  Page 8 |
| 28. | Participant checking | Participant checking  Did participants provide feedback on the findings? | There was no participant checking | N/A |
| *Reporting* | | | | |
| 29. | Quotations presented | Were participant quotations presented to illustrate the themes / findings? Was each quotation identified? e.g. *participant number* | Yes. Each quote contains a participant number | Results  Page 11-18 |
| 30. | Data and findings consistent | Was there consistency between the data presented and the findings? | Yes | Results  Page 8-18 |
| 31. | Clarity of major themes | Were major themes clearly presented in the findings | Yes | Results  Page 8-18 |
| 32. | Clarity of minor themes | Is there a description of diverse cases or discussion of minor themes? | No | N/A |

## Appendix 4 sub analysis

**Age**

| N= | 160 |
| --- | --- |
| Mean | 38 |
| Median | 36 |
| Minimum | 17 |
| Maximum | 65 |

| Age | **Frequency** | **Percent** |
| --- | --- | --- |
| 17- 36 years | 79 | 49,4 |
| >36-65 years | 81 | 50,6 |
| Total | 160 | 100 |

|  | **Independent samples test Levenes Test** | **Cohen’s d** | **Mann-Whitney test** | **Anova/regression** |
| --- | --- | --- | --- | --- |
| 1. Watching the VR movie has made me more aware of what an older adult experiences during the rehabilitation process | t; -0,651  P; 0,516 | Cohen’s d; -0,103 | U; 2824,500  Z; - 1,275  P; 0,202 | R2; 0,000  P; 0,956  Beta; 0,004 |
| 2. I would preceive the care received in the film as pleasant | t; 1,940  P; 0,054 | Cohen’s d; 0,312 | U; 2526,0  Z; -1,793  P; 0,073 | R2; 0,010  P; 0,212 > 0,05  Beta; - 0,101 |
| 3. VR is an effective tool for enhancing my personal awareness/  consciousness | t; -0,599  P; 0,550 | Cohen’s d; - 0,095 | U; 3041,0  Z; -0,307  P; 0,759 | R2; 0,006  P; 0,861  Beta; 0,014 |
| 4. I would recommend this VR movie to my colleagues | t; -1,379  P; 0,170 | Cohen’s d; -0.222 | U; 2675,0  Z; -1,326  P; 0,185 | R2; 0,004  P; 0,462  Beta; 0,060 |
| 5. Overall appreciation | t; -1,036  P; 0,302 | Cohen’s d; -1,167  Klein effect | U; 2687,5  Z; -1,069  P; 0,285 | R2; 0,005  P; 0,367  Beta; 0,073 |

**Years of professional experience**

| N= | 115 (45 missing) |
| --- | --- |
| Mean | 8,88 |
| Median | 4 |
| Minimum | 0 |
| Maximum | 35 |

|  | **Frequency** | **Percent** |
| --- | --- | --- |
| 0-4 years | 59 | 36.9 |
| 4-35 years | 56 | 35 |
| Total | 115 | 71.9 |
| Missing | 45 | 28.1 |
| Total | 160 | 100 |

|  | **Independent samples test Levenes Test** | **Cohen’s d** | **Mann-Whitney test** | **Anova/regressie** |
| --- | --- | --- | --- | --- |
| 1. Watching the VR movie has made me more aware of what an older adult experiences during the rehabilitation process | t; -0,116  P; 0,908 | Cohen’s d; -0,022 | U; 1538,5  Z; -0,527  P; 0,598 | R2; 0,002  P; 0,628  Beta; 0,046 |
| 2. I would preceive the care received in the film as pleasant | t; 1,126  P; 0,262 | Cohen’s d; 0,214 | U; 1372,5  Z; -1,046  P; 0,296 | R2; 0,022  P; 0,122  Beta; - 0,148 |
| 3. VR is an effective tool for enhancing my personal awareness/  consciousness | t; -0,331  P; 0,741 | Cohen’s d; -0,62 | U; 1483,5  Z; -0,718  P; 0,473 | R2; 0,007  P; 0,376  Beta; 0,084 |
| 4. I would recommend this VR movie to my colleagues | t; 1,115  P; 0,267 | Cohen’s d; 0,211 | U; 1438,0  Z; -0,874  P; 0,382 | R2; 0,001  P; 0,710  Beta; 0,036 |
| 5. Overall appreciation | t; -0,596  P; 0,553 | Cohen’s d; -0,113 | U; 1426,0  Z; -0,895  P; 0,371 | R2; 0,004  P; 0,497  Beta; -0,065 |

**Professional role**

|  | **Frequency** | **Percent** |
| --- | --- | --- |
| Nurse | 53 | 33.1 |
| Paramedics | 54 | 33.8 |
| Manager | 6 | 3.8 |
| Health care assistant | 11 | 6.9 |
| Physician | 11 | 6.9 |
| Facility staff | 11 | 6.9 |
| Other | 14 | 8.8 |
| Total | 160 | 100 |

|  | **One way anova** | **Homogeneity of variance** |
| --- | --- | --- |
| 1. Watching the VR movie has made me more aware of what an older adult experiences during the rehabilitation process | F; 0,638  P; 0,700 | Levene; 0,707  P; 0,644 |
| 2. I would preceive the care received in the film as pleasant | F; 0,629  P; 0,707 | Levene; 0,646  P; 0,693 |
| 3. VR is an effective tool for enhancing my personal awareness/  consciousness | F; 1,064  P; 0,138 | Levene; 1,646  P; 0,138 |
| 4. I would recommend this VR movie to my colleagues | F; 1,619  P; 0,146 | Levene; 0,863  P; 0,523 |
| 5. Overall appreciation | F; 0,799  P; 0,572 | Levene; 0,338  P; 0,916 |

## Appendix 5 Code-tree of the focus groups

During the coding of the focus group data, a distinction was made between reflections from the perspective of HCPs imagining themselves as the older adult, and reflections on their own professional actions as shown in the film. Within this distinction, four themes emerged

- Feeling overwhelmed
- Being dependent and vulnerable
- Lack of clarity in staff routines
- Practical improvement in daily care

There is the older adults' perspective as represented in the VR movie, interpreted by HCPs

- Dependent
- Having nothing to say
- Talked over
- Overwhelming
- Family and informal caregiver
- Coping
- Uncertainty
- Restlessness
- Putting oneself in someone else’s shoes
- Awareness
- Feeling like just a number

There are the HCPs' own reflections on their practice following the immersive experience

- Our current situation
- Comparison with the ideal situation
- Importance of a do not disturb sign
- Habituation of healthcare professionals
- Educational use
- Expectations
- Transparency
- Space for the patient
- Personal approach
- Information about
- Explaining what you are going to do
- Balancing needs and care
- Communication
